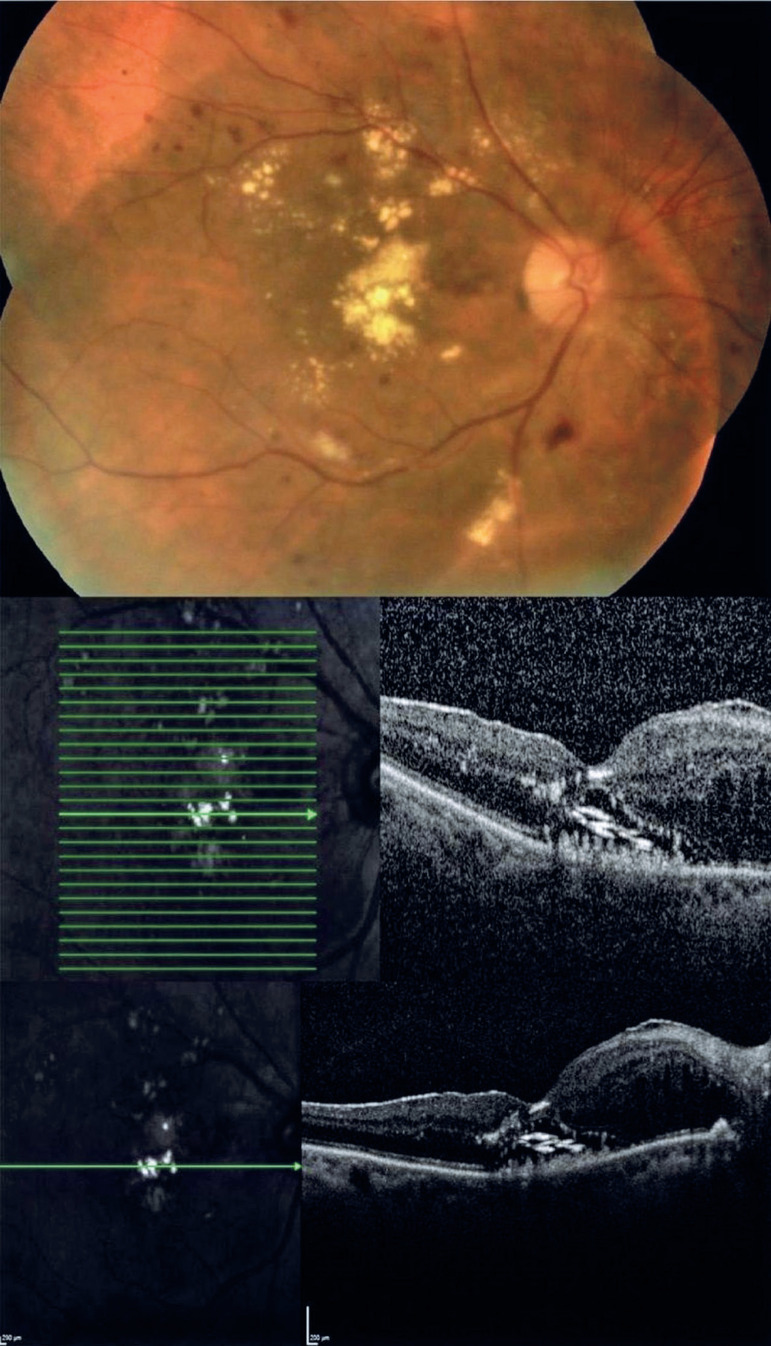# Appearance of an unusual sign on optical coherence tomography in diabetic maculopathy: subretinal onion rings

**DOI:** 10.5935/0004-2749.2024-0172

**Published:** 2024-12-18

**Authors:** Kemal Tekin, Merve Inanc, Cemile Ucgul Atilgan

**Affiliations:** 1 Ophthalmology Department, Ulucanlar Eye Training and Research Hospital, Ankara, Turkey

A 65-year-old female patient with type 2 diabetes mellitus and hypercholesterolemia presented with moderately graded nonproliferative diabetic retinopathy with widespread hard exudates (HEs) in the macula (see the top of the figure). The HEs appeared as golden--yellow lesions on the fovea. In addition, pale-yellow HEs were detected in regions temporal and superior to the fovea, surrounding the foveal lesions (see the top of the figure). Optical coherence tomography (OCT) of the fovea showed serous macular detachment and nasally cystoid macular edema, in addition to an unusual finding of intensely hyper-reflective horizontal deposits over the retinal pigment epithelium without posterior shadowing; these were termed “onion ring signs” (see the middle bottom of the figure).

In patients with macular neovascularization, cholesterol crystals are seen as hyper-reflective horizontal deposits in the subretinal pigment epithelium-basal laminar space, which appear as highly intense signals without any shadowing on OCT^(^1^,^2^)^. Venkatesh et al.^(^3^)^ also used OCT and found onion ring signs in patients with diabetic retinopathy.

**Figure:**